# Raman Spectroscopy Combined with Machine Learning Reveals Myalgic Encephalomyelitis–Associated Biomolecular Signatures at Rest and After Standardized Stress

**DOI:** 10.3390/ijms27114937

**Published:** 2026-05-29

**Authors:** Maryam Heidarifard, Atefeh Moezzi, Frédérick Dallaire, Katherine Ember, Wesam Elremaly, Iurie Caraus, Anita Franco, Frédéric Leblond, Alain Moreau, Mathieu Dehaes

**Affiliations:** 1 Azrieli Research Center, CHU Sainte-Justine, Montreal, QC H3T 1C5, Canada; 2 Research Center, CHUM, Montreal, QC H2X 0A9, Canada; 3 Institute of Biomedical Engineering, Université de Montréal, Montreal, QC H3T 1J4, Canada; 4 Department of Biochemistry and Molecular Medicine, Faculty of Medicine, Université de Montréal, Montreal, QC H3T 1J4, Canada; alain.moreau.hsj@ssss.gouv.qc.ca (A.M.); 5 Viscogliosi Laboratory in Molecular Genetics of Musculoskeletal Diseases, Azrieli Research Center, CHU Sainte-Justine, Montreal, QC H3T 1C5, Canada; 6 Open Medicine Foundation ME Collaborative Center, CHU Sainte-Justine/Université de Montréal, Montreal, QC H3T 1C5, Canada; 7 ICanCME Research Network, Azrieli Research Center, CHU Sainte-Justine, Montreal, QC H3T 1C5, Canada; 8 Department of Engineering Physics, Polytechnique Montreal, Montreal, QC H3T 0A3, Canada; 9 Department of Stomatology, Faculty of Dentistry, Université de Montréal, Montreal, QC H3T 1J4, Canada; 10 Department of Radiology, Radio-Oncology and Nuclear Medicine, Université de Montréal, Montreal, QC H3T 1J4, Canada

**Keywords:** myalgic encephalomyelitis, post-exertional malaise, label-free Raman spectroscopy, machine learning modeling, blood plasma, biomarkers

## Abstract

Myalgic encephalomyelitis (ME) is characterized by profound fatigue, post-exertional malaise (PEM), and cognitive dysfunction. Despite its clinical significance, the pathophysiology of PEM and disease heterogeneity remain unclear, and no validated biomarkers are available for rapid diagnosis or monitoring. We aimed to develop a screening approach combining label-free Raman spectroscopy (RS) and machine learning modeling (ML) to detect biomolecular changes in blood plasma and differentiate patients with ME from sedentary healthy controls. Blood plasma was collected from 115 patients with ME and 45 controls at rest (T0) and 90 min after a standardized, non-invasive stress test designed to induce PEM. Plasma samples were analyzed by RS, and ML models were developed independently at each time point to differentiate patients with ME and controls. The RS-ML models identified spectral features consistent with contributions from proteins, lipids, and low-molecular-weight metabolites. At T0 and T90, the area under the receiver operating characteristic curve, accuracy, sensitivity and specificity were 0.85 and 0.83, 79% and 84%, 82% and 90%, and 73% and 69%, respectively. RS-ML provides a rapid, low-cost approach to detect ME-associated biomolecular signatures in plasma and capture biochemical alterations associated with standardized stress.

## 1. Introduction

Myalgic encephalomyelitis (ME), also referred to as chronic fatigue syndrome, is a complex, multisystem, chronic illness of unknown etiology that affects approximately 600,000 Canadians and up to 2.5 million Americans [1,2], with a global prevalence estimated at 0.4–2.5%. Clinically, ME is characterized by unexplained fatigue persisting for at least six months, sleep disturbances, orthostatic intolerance, cognitive impairment, and post-exertional malaise (PEM) [3,4]. PEM is the cardinal feature of the disease and is characterized by symptom exacerbation following minimal physical or mental exertion; however, its underlying molecular mechanisms remain poorly understood [5]. Given the highly dynamic and heterogeneous nature of ME, clinical characterization alone may be insufficient to fully elucidate disease-related molecular processes [6]. Accordingly, monitoring disease progression and evaluating temporal biomolecular changes in response to PEM are clinically relevant. A rapid, non-invasive, and label-free approach for identifying biomolecular changes could provide valuable insights for early diagnosis and the development of novel therapeutic strategies.

The pathophysiology of PEM remains poorly understood; however, metabolic disturbances and immune dysregulation have been described following exercise [7]. Although a majority of patients with ME report a prodromal illness consistent with infection, no single infectious agent has been consistently implicated [8]. These prodromal infections are frequently linked to viral triggers such as Epstein–Barr virus [9], beta coronaviruses associated with severe acute and Middle East respiratory syndromes [10,11], and COVID-19 (often referred to as “Long-COVID”) [7,12]. The lack of convergence in identifying a specific causative agent suggests that shared host responses to multiple microbial triggers may play an important role in disease pathogenesis, and that ME and related post-infectious syndromes may represent a broader clinical category.

Raman spectroscopy (RS) is a light-based, label-free technique used to assess the biomolecular composition (e.g., proteins, amino acids, nucleic acids, and lipids) of a sample [13]. RS is a non-destructive method based on the excitation of molecules by light illumination and the determination of their molecular vibrational modes via detection of scattered light [14,15]. RS is a rapid and relatively low-cost technique, making it suitable for potential clinical implementation. Previous studies have utilized single-cell Raman microspectroscopy to differentiate patients with ME from controls in peripheral blood mononuclear cells [16,17,18]. However, this technique is costly, low-speed, and unsuitable for large-scale testing required for broad clinical implementation.

Previous studies have combined RS with machine learning (ML) modeling to enhance ME diagnosis, achieving accuracies up to 91% [16,17,18,19,20,21]. To our knowledge, RS-ML has not previously been applied to classify patients with ME and healthy controls in response to a standardized post-exertional stress test and identify potential disease-specific biomarkers associated with PEM. In this study, we developed a RS-ML screening tool to identify biomolecular changes in blood plasma at rest and in response to PEM in patients with ME, and to differentiate them from controls. We hypothesized that this methodology could capture biomolecular perturbations associated with chronic inflammation and PEM in ME, thereby supporting the development of targeted therapeutic strategies.

## 2. Results

### 2.1. Clinical and Demographic Variables of Study Participants

The clinical and demographic characteristics of study participants are summarized in Table 1. The study cohort included 115 patients with ME (100 females/15 males) and 45 sedentary healthy controls (28 females/17 males) who were recruited prospectively and consecutively prior to the COVID-19 pandemic. Age and sex differed between patients with ME and healthy controls (HC). Mental and physical scores from the short-form health survey (SF-36) questionnaires were lower in patients with ME compared to HC. All domains from the Multidimensional fatigue inventory-20 (MFI-20) were higher in patients with ME compared to HC. Compared to HC, patients with ME showed higher DePaul symptom questionnaire (DSQ) scores. The median illness duration among patients with ME was seven years (IQR: 3–16), indicating a chronically affected population.

### 2.2. RS-ML Modeling Performance and Extracted Spectral Features

Two RS-ML models were developed to classify patients with ME and HC at baseline (T0) and at 90 min following the post-exertional stress test (T90). Biomolecular assignments of spectral features extracted by these models were made based on literature values [22,23,24,25,26,27,28,29,30,31,32,33,34,35] and summarized in Table 2 with peak centers and ranges. The performance of these two models is summarized in Table 3: the model at T0 achieved an area under the receiver operating characteristic curve (AUC) value of 0.85, an accuracy of 79%, a sensitivity of 82% and a specificity of 73%, while the model at T90 demonstrated an AUC value of 0.83, an accuracy of 84%, with a sensitivity of 90% and a specificity of 69%.

**Figure 1:**
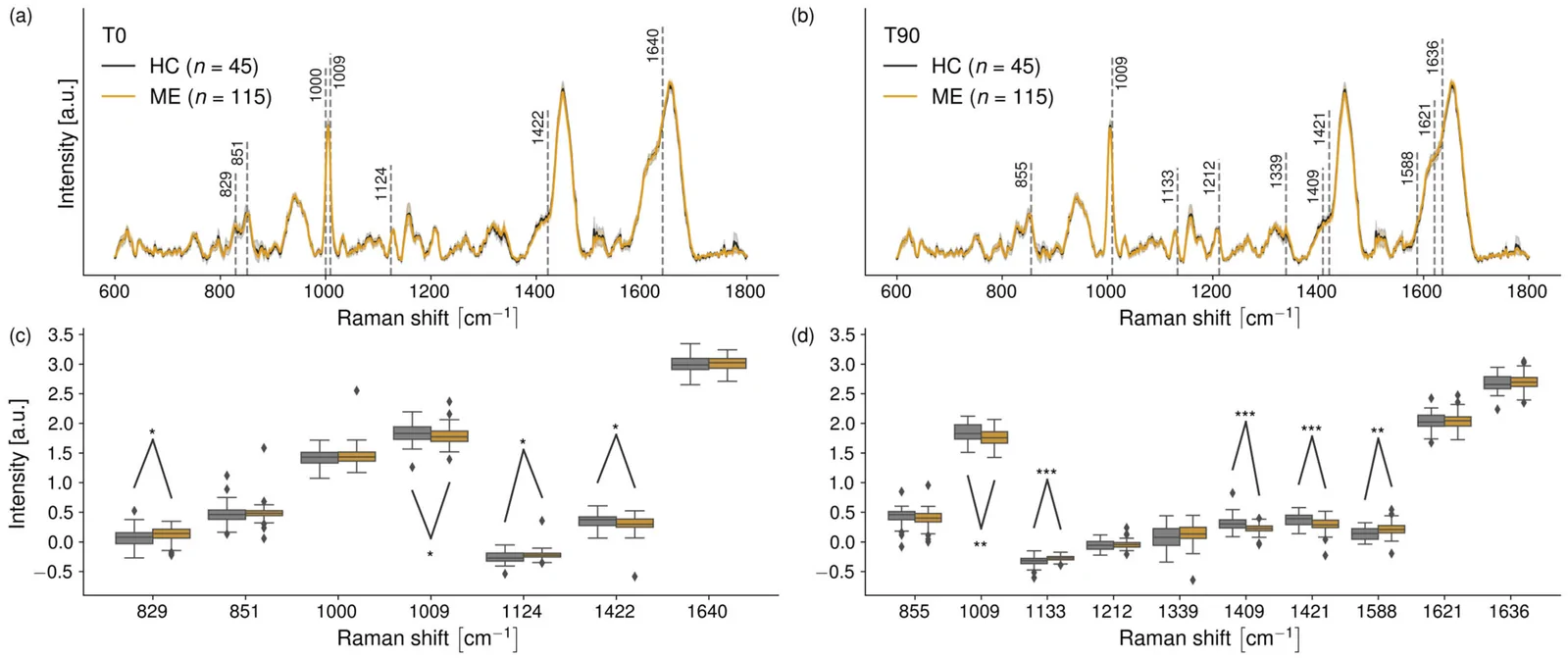
Classification of patients with myalgic encephalomyelitis (ME) and healthy controls (HC) at rest (T0) and 90 min following the post-exertional stress test (T90). Mean Raman spectra with extracted features highlighted by vertical lines for patients with ME (*N* = 115; orange) and healthy controls (*N* = 45; gray) at T0 (**a**) and at T90 (**b**). Box-and-whisker plots showing the relative intensities of Raman features extracted by the machine learning model at T0 (**c**) and T90 (**d**). * *p* < 0.05, ** *p* < 0.01, *** *p* < 0.001.

**Figure 2:**
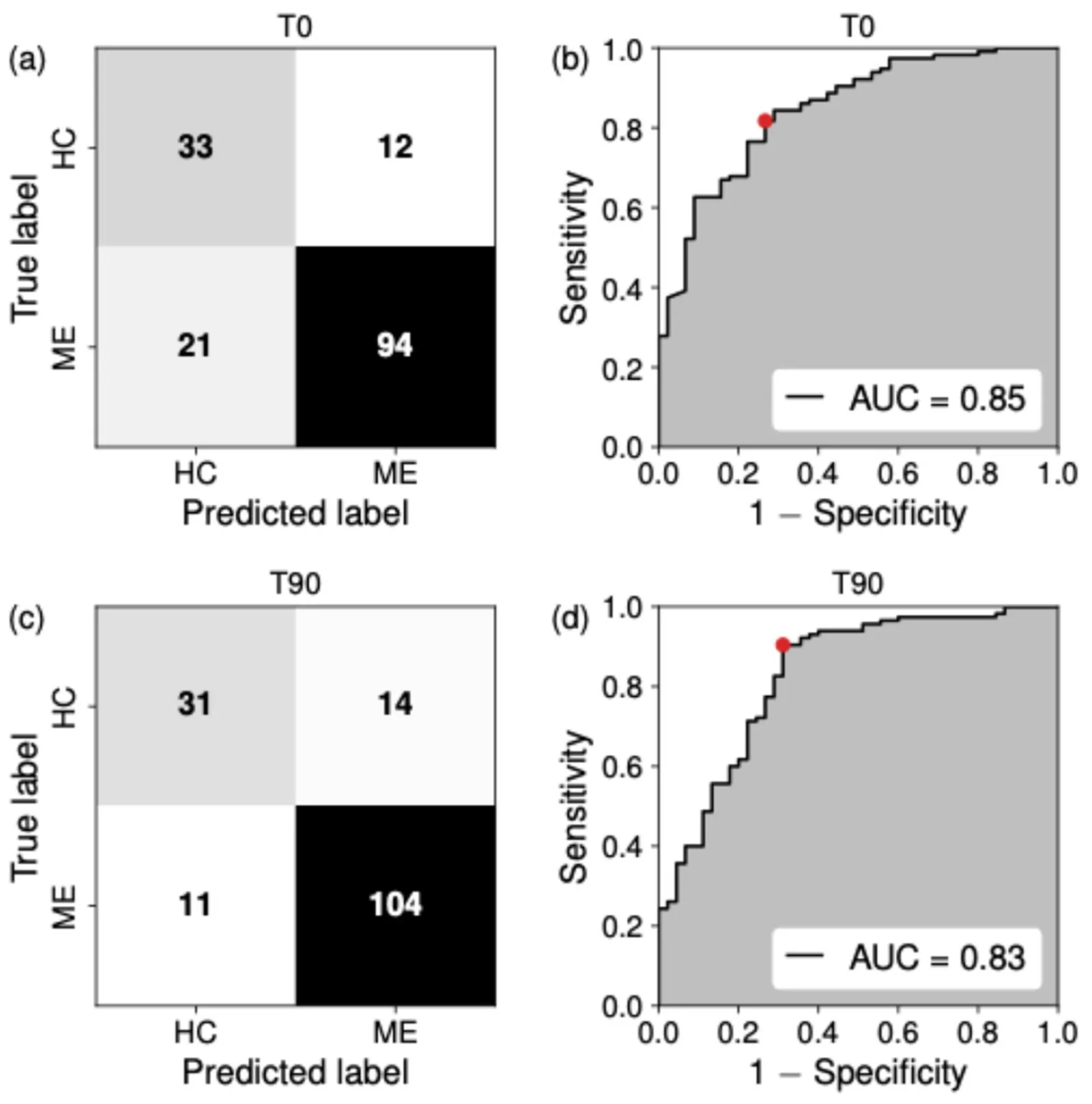
Classification of patients with myalgic encephalomyelitis (ME) and healthy controls (HC) at rest (T0) and at 90 min following the post-exertional stress test (T90). Confusion matrix and area under the receiver operating characteristic curve (AUC) at T0 (**a**,**b**) and T90 (**c**,**d**), respectively. The red dot corresponds to the classification threshold used to maximize the difference between the true and false positive rates (Youden’s J Statistic).

For each RS-ML model, mean Raman spectra are presented with the extracted key features highlighted by vertical dotted lines (Figure 1a,b). The distribution of the intensity values of the Raman peaks extracted by each model are illustrated in Figure 1c,d. Confusion matrices are shown in Figure 2, with the corresponding AUC value, along with sensitivity and specificity.

At T0, Raman features were localized at 829, 851, 1000, 1009, 1124, 1422, and 1640 cm^−1^. Compared with HC, Raman signal intensities corresponding to proteins, lactic acid, and citrate (829 cm^−1^), as well as fatty acid, lipid trans-conformation (C=C), proteins, and glucose (1124 cm^−1^) were increased in patients with ME, whereas the peaks at 1009 (protein [phenylalanine, tryptophan] and urea) and 1422 cm^−1^ (lipids in the form of CH_2_) were reduced. In addition, an increased signal in the peak at 1124 cm^−1^ was observed in patients with ME, reflecting contributions from both proteins, lipids, fatty acids, and/or glucose.

The spectral features selected by the model at T90 differed partially from those identified at baseline and were centered at 855, 1009, 1133, 1212, 1339, 1409, 1421, 1588, 1621, and 1636 cm^−1^. Compared with HC, patients with ME showed increased Raman signal intensities at 1133 cm^−1^ (fatty acid, lipid trans-conformation [C=C], proteins, and glucose) and 1588 cm^−1^ (proteins [amide II]) whereas the phenylalanine/tryptophan (1009 cm^−1^) and CH_2_-lipids (1409 and 1421 cm^−1^) peaks were reduced.

## 3. Discussion

ME remains a diagnostically challenging condition, largely due to the absence of rapid, objective, and scalable biomarkers capable of capturing its complex and dynamic biology. Current diagnostic approaches rely on symptom-based criteria, which limits early detection, patient stratification, and clinical monitoring. There is therefore a critical need for technologies that are not only biologically informative but also label-free, cost-effective, and compatible with real-world clinical implementation.

In this study, we demonstrate that a RS-ML approach applied directly to plasma samples can discriminate patients with ME from healthy controls with consistent performance at rest and following a standardized stress challenge. The RS-ML models identified reproducible spectral patterns reflecting contributions from proteins, lipids, and low-molecular-weight metabolites, indicating that this label-free approach can capture systemic biochemical alterations associated with ME using very low volumes of plasma. Importantly, these Raman-derived features represent integrated biochemical signatures rather than direct measurements of individual metabolites, consistent with the composite nature of Raman signals in complex biofluids.

A key contribution of this work lies in the integration of a controlled stress paradigm designed to induce PEM, a defining feature of ME. While most biomarker studies focus on resting conditions, this approach enables the simultaneous interrogation of baseline biochemical alterations and dynamic responses to physiological stress at the earliest stage possible. Modeling performance at T90 allowed to reduce the number of false negative by half compared with T0. Also, the identification of spectral features that are preserved across baseline and post-exertional conditions suggests the presence of stable, trait-like abnormalities in ME. In contrast, the modulation of specific features following exertion, including the protein-related Raman peaks at 1207, 1339, 1593, and 1625 cm^−1^, supports the existence of stress-sensitive, state-dependent biochemical responses. In addition, the peak at 827 cm^−1^ originally extracted at rest was not selected by the model at T90. This dual profiling framework provides a functional dimension to biomarker development and may enhance disease specificity, as immediate changes leading to PEM represent a clinically distinctive trait of ME.

The 1005 cm^−1^ Raman band is commonly associated with phenylalanine ring breathing modes and may also include contributions from other plasma constituents such as urea and protein-associated vibrations. The decreased intensity of this band in patients with ME at both time points suggests alterations in protein-related or low-molecular-weight metabolite signatures. However, given the composite nature of Raman spectral features in plasma, these signals should be interpreted as reflecting integrated biochemical changes rather than direct quantification of specific metabolites. Other selected features at 1129, 1207, 1339, and 1416 cm^−1^ fall within spectral regions typically associated with C–C, C–N, and CH_2_ vibrational modes, which may reflect contributions from lipids, amino acids, and protein backbones. Similarly, the bands at 1593 and 1625 cm^−1^ are located within regions commonly attributed to aromatic amino acids and amide-related vibrations, suggesting potential alterations in protein conformation or composition. These assignments remain putative due to the overlapping contributions of multiple biomolecular species in plasma. Notably, while some spectral regions were also selected by the model at baseline, their discriminatory contribution differed between time points. These features (1005, 1129 and 1416 cm^−1^) reached stronger statistical significance at T90, indicating that their relevance for group separation may be enhanced under post-exertional conditions. These observations support the notion that standardized stress may modulate disease-associated biochemical signatures detectable in plasma.

The spectral signatures identified in this study are consistent with prior evidence of metabolic dysregulation in ME. Protein-related Raman features associated with amino acid residues—including phenylalanine, tryptophan, tyrosine, alanine, leucine, lysine, proline, and serine—align with previous reports of altered amino acid metabolism and its role in mitochondrial function, immune regulation, and energy homeostasis [36]. Metabolomic studies have consistently reported disturbances in amino acid pathways in ME, including alterations linked to impaired energy production and lipid metabolism [3,37,38]. In addition, changes in circulating amino acids involved in neurotransmission may contribute to cognitive dysfunction and “brain fog” frequently reported by patients [38,39].

The more pronounced difference between groups at the 1005 cm^−1^ Raman peak following exertion further supports the involvement of aromatic amino acid metabolism in ME [22,23,25]. In particular, dysregulation of tryptophan metabolism and the kynurenine pathway have been implicated in neuroinflammation and cognitive impairment [17,36,40]. These findings suggest that physiological stress may exacerbate underlying metabolic vulnerabilities, providing a biochemical basis for PEM and related symptoms [41]. This peak is also assigned to urea, which was lower in patients with ME compared with controls at T90, potentially indicating exertion-associated alterations in nitrogen metabolism in ME. Previous studies have shown that dysregulation of the urea cycle, protein synthesis, and xenobiotic metabolism in ME is associated with persistent cellular stress and fatigue [42,43].

Alterations in protein structure and composition are also suggested by the persistent extraction of Raman features at 851, 1005, 1129, and 1640 cm^−1^ across both time points. These signals, associated with protein backbone and amino acid side-chain vibrations, likely reflect global changes in protein organization rather than isolated metabolite shifts. Their persistence at T90 suggests that exertion may further modulate protein-related biochemical states associated with fatigue and PEM [44].

Lipid metabolism plays a critical role in ME through its involvement in energy production, immune regulation, and mitochondrial function [45]. In the present study, Raman signal intensity in the 1129 cm^−1^ region (fatty acid, unsaturated fat [C=C], and glucose) was increased in patients with ME compared with controls at rest and remained elevated following exertional stress. In contrast, saturated lipids (CH_2_ form, 1416 cm^−1^) were decreased in patients with ME compared with controls at both time points. Consistent with these findings, previous studies have reported dysregulated fatty acid metabolism in ME, which correlates with pain severity and cognitive dysfunction [46]. Such dysregulation has also been linked to mitochondrial and peroxisomal impairments, which can lead to triglyceride accumulation due to reduced peroxisomal activity [47]. Patients with ME also frequently exhibit impaired glucose regulation, presenting as hypoglycemia due to adrenal dysfunction or as hyperglycemia and insulin resistance, both of which contribute to disrupted energy homeostasis [48]. Altered glucose metabolism and elevated lactate levels are key metabolic features of ME and have been implicated in muscle pain, fatigue, and cognitive impairment [49]. These abnormalities may be driven in part by mitochondrial dysfunction, which forces cells to rely on less efficient modes of energy production [50]. Additionally, dysregulated astrocyte-mediated energy metabolism may contribute to glycogen depletion in brain and muscle, thereby contributing to fatigue and unrefreshing sleep [51,52].

Citric acid (citrate), an important intermediate in the tricarboxylic acid (TCA) cycle, provides additional insight into mitochondrial energy metabolism in ME [53]. Previous studies have reported elevated citrate levels after exercise in ME, suggesting disruption of the TCA cycle, impaired mitochondrial energy metabolism, and altered lipid metabolism [43,54]. Within this context, the Raman feature extracted at 827 cm^−1^ (tyrosine, lactic acid and citrate) along with contributions from low-molecular-weight metabolites may reflect broader disturbances in energy metabolism, although Raman spectroscopy does not allow direct metabolite quantification.

The Raman peak at 1416 cm^−1^, assigned to lipid-related vibrations, was extracted by the RS-ML models at both time points. The persistence of this signal suggests that alterations in lipid metabolism in ME may represent a stable component of the disease phenotype rather than a transient response to exertional stress. Consistent with this interpretation, previous studies have reported lipid dysregulation and chronic energetic strain in patients with ME, with alterations in lipid classes linked to disease pathophysiology [55,56]. In addition, sustained lipid abnormalities may reflect ongoing oxidative stress, which has been implicated in mitochondrial dysfunction and inflammation in ME [57].

Previous studies have demonstrated the potential of Raman spectroscopy in detecting ME in peripheral blood mononuclear cells outside of point-of-care settings [16,17,18]. However, these approaches have not yet met the requirements for rapid and affordable point-of-care analysis. Several limitations contribute to this gap, including reliance on microscope-based Raman systems and sample preparation steps that are incompatible with clinical workflows. In approaches involving dried biofluid samples, sample drying is time-consuming, with a 2 µL droplet requiring up to 30–45 min to dry, and heterogeneous drying can introduce intra-sample variability when the laser interrogates only a small region of the sample. Additionally, the use of high-magnification microscope objectives increases sensitivity to focal distance and sample positioning, potentially leading to variability in signal intensity due to instrument-dependent factors [16,17]. These limitations pose significant barriers to the clinical applicability and point-of-care translation of these Raman-based diagnostic tools.

The baseline classification capability is particularly relevant for clinical implementation, as it supports potential applications in patient triaging, prognosis, and treatment planning in a rapid (<3 min) and reagent-free manner. Clinically, the combined analysis of baseline and post-exertional states may provide complementary information on both underlying disease biology and physiological responses to stress. Given that ME is associated with an estimated annual economic burden of up to $51 billion in the United States [43], the development of scalable and accessible diagnostic tools remains a priority. However, for screening applications, particularly in low-prevalence populations, high specificity will be required. Further validation in larger and more diverse cohorts will therefore be necessary to improve model performance and to assess disease specificity relative to related conditions such as fibromyalgia or multiple sclerosis. The RS system used in this study requires only a low volume of plasma and is portable and user-friendly. It can be placed on a small cart within an enclosure, allowing easy transport to hospitals, clinics, or pharmacies. The overall cost of the system is lower than that of commercially available Raman microscopes. Moreover, the results presented here open the door for the future integration of RS-ML into a rapid, point-of-care, plasma-based screening and monitoring tool for ME, including potential assessment of PEM severity in housebound or bedbound patients, and extension to related conditions such as fibromyalgia and Long-COVID syndromes.

While our RS-ML approach demonstrates promising potential as a rapid and scalable tool for ME classification, this pilot study has several limitations. There was an imbalance between the number of males and females, which is due to the higher prevalence of ME in women and the consecutive nature of the recruitment. However, analyses were adjusted for sex to account for this potential confounding factor. The use of a single post-exertional time point at 90 min may not fully capture the temporal dynamics of PEM, particularly in patients exhibiting delayed or prolonged responses. Future studies should incorporate longitudinal sampling strategies and stratify patients according to PEM severity using validated instruments such as the DePaul Post-Exertional Malaise Questionnaire [58,59]. The control cohort size was relatively modest, which may have influenced model performance and generalizability; expanding the control population will be important to improve robustness and refine classification thresholds. Although RS-ML successfully differentiated patients with ME from healthy controls at baseline, external validation in independent cohorts is required to confirm reproducibility and to assess disease specificity relative to clinically overlapping conditions such as fibromyalgia or multiple sclerosis. This pilot study provided AUC values of 0.83–0.85 with accuracies of 0.79–0.84, which is promising but modest for a clinical diagnostic. However, a future study including a larger population would potentially increase the modeling performance and therefore limit the number of false positive and negative cases. RS measurements from the same sample were acquired at a single spot, which may not represent the complete molecular composition of the sample. However, blood plasma lacks large elements such as cells which reduce homogeneity and can sediment out, as occurs in whole blood [60]. Prior to measurements, the plasma samples were vortexed to increase molecular motion, which ensured that the spectra captured molecules diffusing through the sample in different conformations and spatial distributions. The asymmetric nature of some Raman peaks may also complexify data interpretation: while 1000 and 1009 cm^−1^ were extracted at T0 and associated with the same peak (centered at 1005 cm^−1^), only data at 1009 cm^−1^ significantly differed between groups. Finally, while Raman spectroscopy enables rapid, label-free profiling of plasma, its spectral features represent composite biochemical signatures and do not provide direct quantification of individual metabolites, which should be considered when interpreting mechanistic associations.

## 4. Materials and Methods

### 4.1. Study Design and Participants

Patients who met the standard Canadian Consensus Criteria (CCC) for ME diagnosis were consecutively recruited. Clinical variables and demographics were collected through medical records. The study was approved by the Ethics Review Board of the Centre de recherche Azrieli du CHU Sainte-Justine (Montreal, QC, Canada; #4047). Written informed consent was obtained from all participants. Exclusion criteria for healthy controls included any family history of ME or related conditions such as fibromyalgia or multiple sclerosis [61].

### 4.2. Questionnaires

Patients with ME and controls completed three validated, self-reported standardized questionnaires prior to undergoing the stress test: the MFI-20, the SF-36 and the DSQ. The SF-36 was used to assess both physical and mental health, while the MFI-20 evaluated fatigue across five dimensions: general fatigue, physical fatigue, mental fatigue, reduced activity, and reduced motivation [62]. The total MFI-20 score (0 to 100) classified participants into two categories: mild to moderate (51–85) and severe (86–100) [63]. The DSQ assessed the core symptom profile of ME, categorizing responses across four domains: autonomic and neuroendocrine-immune dysfunction, cognitive dysfunction, sleep disturbances, and PEM [64]. Collectively, these questionnaires provided a comprehensive clinical evaluation of symptom burden and health-related quality of life in participants.

### 4.3. Post-Exertional Stress Challenge

To safely elicit PEM in participants, a post-exertional stress challenge was conducted using a therapeutic massage device (ABR therapeutic massager device, Panacis Medical Ltd., Ottawa, ON, Canada). This validated method is non-invasive and employs an inflatable cuff placed on the arm to deliver pulsatile compressions with variable amplitudes (0–4 psi at 0.006 Hz) [58]. The stimulation period was short (90 min) and enabled a rapid and immediate assessment of molecular responses to PEM.

### 4.4. Sample Collection

Blood samples from participants were collected at T0 and 90 min following the post-exertional stress test in EDTA-coated tubes. Plasma was separated by centrifugation at 216× *g* for 10 min at room temperature. The isolated plasma was aliquoted and stored at −80 °C to prevent degradation before analysis.

### 4.5. Raman Spectroscopy System, Data Acquisition and Preprocessing

The RS system was based on a 785 nm excitation wavelength and captured inelastically scattered light within the spectral range of 600–1800 cm^−1^. Light was emitted using a 100 mW laser (IPS, Plainsboro, NJ, USA), providing high signal-to-noise ratio (SNR) while avoiding sample degradation. The collected light was analyzed with a spectrometer (Wasatch, Morrisville, NC, USA) equipped with a 1024-pixel CCD camera (grating of 600 ± 0.5 lines/mm, spectral resolution of 8 cm^−1^) and optimized for high SNR with short exposure times. Short integration times (300–1000 ms per measurement) were used to enable equivalent Raman signal levels between samples. Plasma samples (100 µL) were placed in a 400 µL enhanced chemical resistance aluminum cuvette and positioned at 11 mm from the laser probe to minimize noise and prevent signal contamination from the cuvette walls. Liquid plasma samples were used to provide high Raman spectral accuracy. Before data acquisition, plasma samples were thawed using a controlled procedure: plasma samples were transferred from dry ice to regular ice for 30 min and rested in room temperature for another 30 min. Samples were further vortexed for 60 s to ensure molecular mixing. Spectral data preprocessing steps included: (1) data averaging from 20 measurements performed at a single spot (size at the tip 170 μm, collection area ~1 mm diameter) per sample, (2) cosmic-ray artifact removal, (3) baseline subtraction of the autofluorescence signal using the Bubble Fill algorithm [65], (4) smoothing using a Savitzky–Golay filter of order 3 with a window size of 11 [66], and (5) standard normal variate normalization [67]. At each time point, 160 Raman averaged spectra were considered: 115 for patients with ME and 45 for HC. Codes were developed in Python 3.12.4 software.

### 4.6. Features Selection and Machine Learning Modeling

To reduce the feature space from 1200 to 26, a linear support vector machine (SVM) with L1 regularization was utilized. These 26 universal peaks were included in a library and fitted with Gaussian functions to reduce the spectrum to the full width at half maximum of these peaks. A statistical weight for each peak was provided by the L1-regularized SVM [67]. Features were ranked according to their weight. A gradient boosting model based on decision trees was used to classify participants based on Raman data, age and sex as inputs. Age and sex were included as they were previously identified as risk factors for ME [68]. This approach was selected for its efficiency in handling mixed data types and its ability to capture non-linear relationships, while minimizing overfitting through ensemble averaging and regularization [69]. Hyperparameters optimization was carried out using a grid search approach that ran over various combinations of hyperparameters including class weight, learning rate, number of estimators, minimum loss reduction, L1 and L2 regularization terms, and maximum tree depth. To account for imbalanced sample sizes between patients with ME (*N* = 115) and HC (*N* = 45), the class weight hyperparameter was defined by the ratio 115/45 for the class ME while the class weight equaled 1 for HC. During data training, the penalty term associated with the misclassification of patients with ME was higher than that of HC. A five-fold cross-validation approach was used to assess model classification performance. The datasets were randomly partitioned into five equal-sized folds, with 80% of the data used for training and 20% for testing. To mitigate classification bias and prevent data leakage, samples from the same participant were included exclusively in either the training or the testing set during cross-validation. Model performance was evaluated using the area under the receiver operating characteristic curve, and results are reported for the hyperparameters configuration yielding the highest AUC, together with the corresponding sensitivity, specificity, and accuracy. Confusion matrices were generated and reported alongside the selected Raman features to facilitate model interpretation. Classification was performed using Python 3.12.4 (libraries Xgboost and Scikit Learn).

### 4.7. Statistical Analyses

Variables are reported as median and interquartile range (IQR, 25th–75th percentile) for non-normally distributed continuous variables. Categorical variables are expressed as frequencies and percentages. Student’s *t*-test was used to compare normally distributed continuous variables between groups, while the non-parametric Mann–Whitney U test was used for non-normally distributed continuous variables. Raman spectral features were compared between groups with multivariate regression analyses adjusted for age and sex. Sex between groups was compared using the chi-square test. A comparison at *p* < 0.05 was defined as statistically significant. Statistical analyses were performed using Python 3.12.4.

## 5. Conclusions

In summary, this study shows that a label-free RS-ML approach applied to plasma can identify reproducible biochemical signatures associated with ME at rest and following a standardized stress challenge. At rest and 90 min following the stress test, the AUC, accuracy, sensitivity and specificity were 0.85 and 0.83, 79% and 84%, 82% and 90%, and 73% and 69%, respectively. The observed spectral patterns are consistent with disturbances in amino acid biology, lipid regulation, and energy metabolism that have previously been implicated in ME. By combining rapid acquisition, minimal sample preparation, and scalable instrumentation, this approach represents a promising step toward the development of accessible tools for ME screening and monitoring. With further validation in larger and more diverse cohorts, RS-ML may contribute to improved patient stratification, more objective assessment of disease activity, and a better understanding of the biological mechanisms underlying ME.

## Figures and Tables

**Table 1:** Clinical and demographic characteristics of patients with myalgic encephalomyelitis (ME) and healthy controls (HC).

Characteristics		ME (*N* = 115)	HC (*N* = 45)	*p*-Value
Age ^a^, median (IQR)		45 (39–58)	42 (34–51)	0.0205
Sex, *N* (%)	Male	15 (13)	17 (38)	0.0004
	Female	100 (87)	28 (62)	
Illness duration ^a^, median (IQR), (years)		7 (3–16)	N/A	
BMI ^b^ (kg/m^2^), median (IQR)		25 (22–29)	27 (23–30)	0.1237
36-item short-form health survey (SF-36) scores, median (IQR)	Mental score	45 (27–55)	89 (86–95)	<0.0001
	Physical score	32 (24–39)	92 (86–97)	<0.0001
Multidimensional fatigue inventory-20 (MFI-20) scores, median (IQR)	General fatigue	17 (15–20)	6 (5–9)	<0.0001
	Physical fatigue	16 (14–19)	6 (4–9)	<0.0001
	Reduced activity ^c^	17 (14–18)	5 (4–8)	<0.0001
	Reduced motivation	10 (8–19)	5 (4–8)	<0.0001
	Mental fatigue	16 (13–18)	7 (5–10)	<0.0001
DePaul symptom questionnaire (DSQ) scores, median (IQR)	Autonomic, neuroendocrine and immune dysfunction score ^c^	40 (30–53)	6 (3–12)	<0.0001
	Cognitive dysfunction score ^c^	54 (39–68)	8 (3–16)	<0.0001
	PEM score ^c^	72 (61–82)	5 (4–12)	<0.0001
	Sleep disturbance score ^c^	48 (38–63)	13 (8–21)	<0.0001

Sex, general fatigue, mental and physical fatigue scores, and reduced motivation were available for all patients with ME. Sample sizes were incomplete for the following variables: ^a^ age and illness duration (*N* = 113), ^b^ BMI (*N* = 110), ^c^ autonomic, neuroendocrine, and immune dysfunction, cognitive dysfunction, PEM, and sleep disturbance (*N* = 114). All variables were available for HC. Abbreviations: BMI, body mass index; IQR, interquartile range (25th–75th percentile); PEM, post-exertional malaise. N/A indicates not applicable.

**Table 2:** Raman peaks, centers, ranges, and corresponding biomolecular assignments for spectral features extracted by the models classifying patients with myalgic encephalomyelitis (ME) and healthy controls (HC) at baseline (T0) and at 90 min following the post-exertional stress test (T90).

Extracted Peak (cm^−1^)	Peak Center (cm^−1^)	Peak Range (cm^−1^)	Biomolecular Assignments
Patients with ME vs. HC at T0			
829	827	821–833	Protein (tyrosine), lactic acid, citrate
851	851	843–859	Glycerol, protein (tyrosine, alanine, leucine, lysine, proline)
1000, 1009	1005	998–1012	Protein (phenylalanine, tryptophan) and urea
1124	1129	1122–1136	Fatty acid (C–C), lipid (C=C), protein (C–N, serine), glucose
1422	1416	1408–1423	Lipid [CH_2_]
1640	1654	1633–1677	Lipid (C=C), protein (amide I)
Patients with ME vs. HC at T90			
855	851	843–859	Glycerol, protein (tyrosine, alanine, leucine, lysine, proline)
1009	1005	998–1012	Protein (phenylalanine, tryptophan) and urea
1133	1129	1122–1136	Fatty acid (C–C), lipid (C=C), protein (C–N, serine), glucose
1212	1207	1201–1213	Protein (tyrosine)
1339	1339	1331–1347	Protein (C–H, aspartate, histidine, proline, valine, tryptophan)
1409, 1421	1416	1408–1423	Lipid [CH_2_]
1588	1593	1582–1604	Protein (amide II)
1621	1625	1617–1632	Protein (tyrosine, tryptophan, phenylalanine)
1636	1654	1633–1677	Lipid (C=C), protein (amide I)

Notes: Amino acids listed alongside the proteins may either present freely in the blood plasma or be bound within protein structures. Assignments were made from literature values [22,23,24,25,26,27,28,29,30,31,32,33,34,35].

**Table 3:** Performance of machine learning models classifying patients with myalgic encephalomyelitis (ME) and healthy controls (HC) at baseline (T0) and at 90 min following the post-exertional stress test (T90).

Classification Models	Accuracy (%)	Sensitivity (%)	Specificity (%)	AUC
Patients with ME vs. HC at T0	79	82	73	0.85
Patients with ME vs. HC at T90	84	90	69	0.83

Notes: Model performance was evaluated using accuracy, specificity, sensitivity, and the area under the receiver operating characteristic curve (AUC).

## Data Availability

The original contributions presented in this study are included in the article. Further inquiries can be directed to the corresponding author.

## Abbreviations

The following abbreviations are used in this manuscript:

AUC	Area under the receiver operating characteristic curve
BMI	Body mass index
CCC	Canadian Consensus Criteria
CCD	Charge-coupled device
Cm^−1^	Inverse centimeters (Raman shift unit)
DSQ	DePaul Symptom Questionnaire
DTA	Ethylenediaminetetraacetic acid
Hz	Hertz
IQR	Interquartile range
L1	Lasso (L1) regularization
L1-SVM	L1-regularized support vector machine
L2	Ridge (L2) regularization
ME	Myalgic encephalomyelitis
MFI-20	Multidimensional Fatigue Inventory (20-item version)
ML	Machine learning
PEM	Post-exertional malaise
Psi	Pounds per square inch
RS	Raman spectroscopy
RS-ML	Raman spectroscopy-machine learning
SD	Standard deviation
SF-36	36-item Short-Form Health Survey
SNR	Signal-to-noise ratio
SVM	Support vector machine
T0	Baseline time point (before stress test)
T90	90 min after the post-exertional stress test
TCA	Tricarboxylic acid (cycle)
